# Level of Agreement Between Problem Gamblers’ and Collaterals’ Reports: A Bayesian Random-Effects Two-Part Model

**DOI:** 10.1007/s10899-019-09847-y

**Published:** 2019-04-02

**Authors:** Kristoffer Magnusson, Anders Nilsson, Gerhard Andersson, Clara Hellner, Per Carlbring

**Affiliations:** 1 Centrum för psykiatriforskning, Karolinska Institutet, Norra Stationsgatan 69, 113 64 Stockholm, Sweden; 2 Linköping University, Linköping, Sweden; 3 Stockholm University, Stockholm, Sweden

**Keywords:** Gambling, CSO–gambler agreement, Skewed data, Intraclass correlations, Two-part models

## Abstract

This study investigates the level of agreement between problem gamblers and their concerned significant others (CSOs) regarding the amount of money lost when gambling. Reported losses were analyzed from 266 participants (133 dyads) seeking treatment, which included different types of CSO–gambler dyads. The intraclass correlation coefficients (ICCs) concerning the money lost when gambling during the last 30 days were calculated based on the timeline followback. In order to model reports that were highly skewed and included zeros, a two-part generalized linear mixed-effects model was used. The results were compared from models assuming either a Gaussian, two-part gamma, or two-part lognormal response distribution. Overall, the results indicated a fair level of agreement, ICC = .57, 95% CI (.48, .64), between the gamblers and their CSOs. The partner CSOs tended to exhibit better agreement than the parent CSOs with regard to the amount of money lost, ICC_*diff*_ = .20, 95% CI (.03, .39). The difference became smaller and inconclusive when reports of no losses (zeros) were included, ICC_*diff*_ = .16, 95% CI (− .05, .36). A small simulation investigation indicated that the two-part model worked well under assumptions related to this study, and further, that calculating the ICCs under normal assumptions led to incorrect conclusions regarding the level of agreement for skewed reports (such as gambling losses). For gambling losses, the normal assumption is unlikely to hold and ICCs based on this assumption are likely to be highly unreliable.

## Introduction

Approximately 2% of the Swedish population are considered to be problem gamblers (Public Health Agency of Sweden 2016), while as many as 18% are estimated to be concerned significant others (CSOs) of problem gamblers (Svensson et al. 2013). The impact of gambling-related harm on CSOs in terms of both financial difficulties and psychological distress has been well-documented (Kalischuk et al. 2006). However, while CSOs are clearly severely affected by problem gambling, the extent to which they actually know about the gambling remains largely unknown.

Qualitative studies concerning the experiences of the CSOs of problem gamblers reveal that such persons generally have only a limited awareness of the gamblers’ problems (Dickson-Swift et al. 2005; Holdsworth et al. 2013; Patford 2009). Some CSOs report being completely unaware of relatively far-reaching gambling problems until confronted with severe financial problems stemming from gambling (Holdsworth et al. 2013; Patford 2007, 2009). Other CSOs describe a situation in which gambling has been an almost constant subject of discussion, although the scope of the gambling problems remained unknown to them (Patford 2007, 2009). The interviewed CSOs commonly depict the gambler’s behavior as deceitful, and they state that he or she has frequently lied about the extent and character of his or her gambling (Dickson-Swift et al. 2005; Holdsworth et al. 2013; Patford 2009). A clinical diagnosis of disordered gambling, as defined within the Diagnostic and Statistical Manual of Mental Disorders, 5th Edition (DSM-5), involves nine diagnostic criteria, of which “Lies to conceal the extent of involvement with gambling” is one (American Psychiatric Association 2013, p. 585). Lying about one’s behavior is not unique to disordered gambling, since it may occur in relation to all types of addictions.

While CSOs report a limited retrospective insight into gambling problems, only a few prior studies have correlated this insight with reports from gamblers. An early study by Taber et al. (1987), concerning the outcome of treatment for problem gambling, used reports by CSOs to corroborate gamblers’ own accounts of days spent gambling some 6 months after treatment ended. The results showed a high degree of consistency with regard to the number of days spent gambling (*r* = .82, *df* = 44, *p* < .01), and out of 46 couples, only two differed in terms of whether or not the gambler had abstained from gambling. A study by Hodgins and Makarchuk (2003) on the reliability of self-reported gambling behavior among problem gamblers used collateral reports (*n* = 66) of timeline followback (TLFB) and compared them to gamblers’ reports. When measured as intraclass correlation coefficients (ICCs), the overall level of agreement was found to be fair to good, with the ICCs ranging from .46 to .65. A number of intervention studies have likewise corroborated gamblers’ accounts of their gambling using reports made by collaterals. Diskin and Hodgins (2009) received collateral reports for 51% (*n* = 36/71) of CSOs with ICCs of .32 for money lost and .65 for days spent gambling, while Hodgins et al. (2009) received collateral reports for 68% of CSOs (of the 279 gamblers followed at week 12) with ICCs of .69 for money lost and .53 for days spent gambling. Further, Petry et al. (2006) found significant Spearman correlations between the reports of gamblers and those of CSOs (*n* = 176/231) of .68 for money lost .62 for days spent gambling.

Interestingly, greater attention has been paid to collaterals’ reports in other fields of addiction, particularly regarding alcohol use. In general, collaterals’ reports have been found to be fairly consistent with reports made by the users (Connors and Maisto 2003; Graham and Braun 1999; Laforge et al. 2005; Sobell et al. 1997; Stasiewicz and Stalker 1999), although CSOs do exhibit a tendency to underestimate drinking and drug use (Borsari and Muellerleile 2009; Hagman et al. 2010). Spouses seem able to offer a more accurate estimation of drinking when compared to other CSOs (Sobell et al. 1997). However, gambling does not produce immediate physiological signs (e.g., intoxication) in the same way that alcohol and drug use does, which means that caution must be applied when making comparisons. Problem gamblers are afforded ample opportunity to gamble in secret, which likely renders it more difficult for CSOs to provide accurate estimations of the scope of the gambling (Downs and Woolrych 2010; Holdsworth et al. 2013). For instance, when Makarchuk et al. (2002) adapted the Community Reinforcement and Family Training (CRAFT) approach from focusing on alcohol and drugs to concentrating on problem gambling, a focus group of CSOs assessed that identifying the occurrence of gambling was the biggest challenge. Moreover, McBride and Derevensky (2008) found that problem gamblers, compared to social gamblers, were much more likely to prefer online gambling because it was easier to hide (29% vs. 5%).

The aim of this study was twofold. First, to investigate the level of agreement between the gambler and their CSO in terms of the money lost when gambling, as well as to determine whether the level of agreement is associated with the CSO’s type of relationship to the gambler. Second, to investigate if other response distributions than the normal distribution provide a better fit when modeling the level of agreement with regard to gambling losses.

## Methods

### Participants

The data used in this study were derived from an intervention study (study protocol: Nilsson et al. 2016) investigating the effects of involving CSOs in internet-delivered treatment for problem gambling. The study involves problem gamblers and their CSOs, who could be their spouse, parent, sibling, or friend. The participants were recruited nationwide via the Swedish National Gambling Helpline, by referrals from health-care professionals who were informed about the study, and through advertisements on Facebook and Google. The gamblers had to score 8 or greater on the Problem Gambling Severity Index (PGSI) to be included in the study.^1^

The participants signed up on a website and were asked to provide information about gambling, psychological well-being, and relationship satisfaction, as well as background data, such as age, gender, and employment status. All the information was collected through surveys conducted online. After being enrolled in the study, the participants were randomized as a dyad into one of two treatment conditions: one involving only the gambler and one where both members of the dyad received treatment. The treatments were based on cognitive behavioral therapy, and they were internet-based, with therapist support being provided via email and telephone (c.f., Carlbring and Smit 2008). Data are also included from a pilot study (Nilsson et al. 2018) conducted prior to the randomized controlled trial (RCT). The pilot and the RCT data were pooled, since the recruitment and screening processes were identical, and recruitment into the RCT followed immediately after the completion of the pilot study. The data collection for the pilot study lasted from March 2015 to August 2015, while the RCT data collection lasted between September 2015 and December 2017.

### Measures

During registration, all the gamblers and their CSOs filled out a TLFB report covering the last 30 days. The TLFB instrument is commonly used to collect information about behaviors linked to addiction. Created in order to monitor daily drinking and problem drinking history (Maisto et al. 1979), TLFB has also been adopted for use in other addiction fields, including gambling (Weinstock et al. 2004). The TLFB instrument resembles a calendar, and users are instructed to record the number of days spent gambling and the amount of money lost to gambling each day throughout a 30-day period. The Banff consensus statement (Walker et al. 2006) stipulates that net losses and time spent on gambling are the most important aspects of gambling behavior. Only the baseline TLFB was used, which was answered prior to randomization, so as to avoid the issue of the agreement being influenced by the treatment. The level of agreement on some secondary variables, namely gambling debt and years spent gambling was also investigated.

### Statistical Analysis

**Fig. 1:**
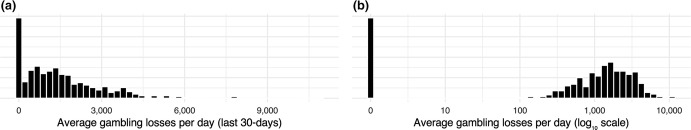
Example of the distribution of gambling losses. **a** Data on the original scale, while **b** Data after a log(y + 1) transformation

ICCs were calculated in order to estimate the level of agreement between the gamblers’ and the CSOs’ TLFB reports. ICCs represent a common method of assessing agreement in dyadic research (Kenny et al. 2006). However, it is important to note that most studies that calculate ICCs rely on a model that assumes a normal (Gaussian) response distribution. This assumption is highly unlikely to hold in the case of gambling losses. Indeed, TLFB data concerning gambling losses are frequently heavily right-skewed, and it is not unexpected to see some participants report losses that are several orders of magnitude above the median. Moreover, the average differences between dyads, as well as the discrepancies within dyads, are both likely to be skewed, and the variance is likely to increase for larger reported losses. A further complication stems from the fact that some participants reported no losses. Figure 1a shows how the reports of gambling losses usually appear, that is, right-skewed, with some persons losing large amounts of money, and some reporting no losses. Figure 1b shows that in the presence of zeros, a log(y + c) transformation (whereby a small constant *c*, is added prior to the transformation in order to get rid of the zeros) does little to remedy the problem; in this paper *c* = 1 was used.

A flexible model that allows for both the inclusion of zeros and the skewed continuous responses is a two-part model^2^ (Neelon et al. Smith 2016; Olsen and Schafer 2001). A two-part model splits the model into two distinct parts, and it models the presence of zeros using a binary model, while it models the actual non-zero losses using a skewed continuous distribution, such as the gamma or lognormal distribution. By including random effects, it is possible to model the variance both within and between the dyads by fitting each part of the two-part model as a two-level generalized linear mixed-effects model (GLMM). This enables a thorough assessment of the level of agreement between gamblers and their CSOs, since ICCs can be calculated for the reported zero losses (binary part), the level of agreement for dyads that report losses (the conditionally positive continuous part of the model), and the overall distribution, which includes reports of both zeros and losses. In addition, the two GLMMs can be linked via correlated random effects (Liu et al. 2010). For the dyads’ reports concerning gambling losses, correlated random effects represents a reasonable assumption, meaning that it is likely that dyads with higher reported losses (continuous part of the model) would exhibit a lower likelihood of reporting a zero (in the binary part of the model). However, in this study, only a small number of participants reported zero losses, which raised the question as to whether including the correlation between random effects would introduce more bias than simply ignoring the correlation. For this reason, results derived from both models are reported, and the models’ performances are assessed using Monte Carlo simulations. It was also investigated whether there were any systematic differences between the gamblers’ and the CSOs’ reports. To allow for comparisons with other gambling studies (e.g., Petry et al. 2006), Spearman’s *rho* is also reported.

### Agreement as a Function of the Type of CSO

In order to investigate whether the level of agreement differed as a function of the type of relationship the CSO had with the gambler (for instance, if they were a parent or a partner), a model was fit wherein both the random effects and the parameters of the GLMM’s distribution family were allowed to be different for each CSO relationship type. This allows for different variances both within and between the dyads as a function of the type of relationship. For the gamma distribution, this meant that the shape parameter was allowed to differ as a function of the relationship type.

### ICCs on the Data Scale

The variance components derived from a GLMM are typically given on the link scale. However, since the focus of this paper was in the level of agreement in the actual TLFB reports, the ICCs were calculated on the observed data scale, which was achieved by calculating the variances both within and between the dyads on the original data scale by integrating over the random effects for all the posterior samples using the methods described in de Villemereuil et al. (2016). The difference between the levels of agreement for the CSOs who were parents or partners could then be compared using the posterior samples by taking the difference.^3^

### Simulation Study

In order to validate the performance of the proposed two-part GLMM in terms of assessing the level of agreement concerning gambling losses, a small Monte Carlo simulation was performed and each simulation involved 5000 replications. Relative bias for the posterior medians were investigated, as well as how well the 95% credible intervals (CI) recovered the true values, under assumptions similar to the data observed in this study. The results were also contrasted the results obtained from a model assuming a Gaussian response distribution, that is, a classical two-level LMM. Furthermore, the two-part model was fit with and without a correlation between the binary and continuous parts of the model, when the true data generating process included a correlation. The focus was on the difference in the estimated ICCs from parents and partners.

### Model Implementation and Software

All computations in this study were performed using R v3.5.1 (R Core Team 2018). The models were fitted using the *brms* v2.4.0 package (Bürkner 2017), which fits models using the Hamiltonian Monte Carlo (HMC) algorithm via the probabilistic programming language Stan (Carpenter et al. 2017; RStan v2.17.3). Uncertainty in the estimates was summarized using the 95% percentile CIs and point estimates by means of the posterior medians. The different models were compared using leave-one-out cross-validation (LOO-CV) or 10-fold CV (Vehtari et al. 2017). The models’ goodness-of-fits were also investigated using posterior predictive checks (Gelman et al. 2014; Gelman et al. 1996), whereby simulation predictions from a fitted model are compared to the observed data in order to see whether any obvious discrepancies can be detected between that fitted model and the observed data.

The Monte Carlo simulations were performed using resources provided by the Swedish National Infrastructure for Computing (SNIC) at the High-Performance Computing Center North (HPC2N), Umeå, Sweden.

An example of the code used to fit the model and run the simulation can be downloaded from the Open Science Framework https://osf.io/ec5b9/.

## Results

### Descriptive Statistics

**Fig. 2:**
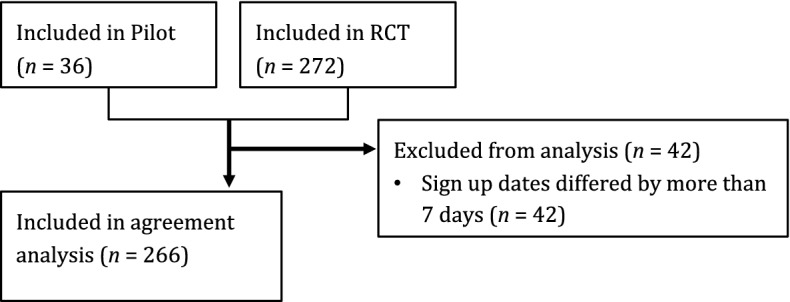
Participant flow

Participants belonging to the same dyad could sign up, and therefore complete the baseline measures, at different times. Since the TLFB instrument is intended to capture losses during the last month, differences in the completion time within the dyads were investigated. In total, 21/154 (14%) of the dyads had an absolute difference of more than 1 week, while 84/154 (55%) of the dyads signed up on the same day. In order to retain as many participants as possible, while still ensuring that the ratings within the dyads covered approximately the same period of time, it was decided to exclude those dyads that had registration dates that differed by more than 7 days; this decision was made prior to analyzing any data (Fig. 2 shows the participant flow). Table 1 presents the background information for the overall sample and split per CSO type.

**Table 1:** Descriptive information for the overall sample and per CSO type

	Full sample (*n* = 133 dyads)		Concerned significant other (CSO) type					
			Partner (*n* = 73, 55%)		Parent (*n* = 50, 38%)		Other (*n* = 10, 7%)	
	CSO	Gambler	CSO	Gambler	CSO	Gambler	CSO	Gambler
Age								
M (SD)	45 (14.5)	35 (11.5)	37.2 (12.6)	38.3 (12.2)	57.7 (7.5)	27 (6.7)	39.0 (10.0)	42 (11.4)
Female								
*n* (%)	103 (77%)	21 (16%)	60 (82%)	12 (16%)	36 (72%)	5 (10%)	7 (70%)	4 (40%)
Days company/week								
M (SD)	5.1 (2.5)	5.2 (2.5)	6.7 (.9)	6.7 (1.1)	3.2 (2.6)	3.5 (2.7)	2.8 (2.1)	2.7 (2.0)
Company 7 days/week								
*n* (%)	78 (59%)	77 (58%)	67 (92%)	63 (86%)	10 (20%)	13 (26%)	1 (10%)	1 (10%)
TLFB confidence (0–100)								
M (SD)	49.1 (31.8)	74 (22)	51.6 (30.7)	76.1 (19.7)	47.8 (33.4)	69.5 (25.6)	37.5 (31.6)	81.5 (15.8)
Average money lost per day (SEK)								
M (SD)	1499 (3392)	1381 (2764)	1658 (4218)	1672 (3561)	1147 (1715)	999 (1271)	2103 (3010)	1168 (511)
Median (IQR)	500 (1304)	552 (1216)	393 (1179)	463 (1266)	500 (1238)	535 (943)	834 (1381)	1164 (429)
Skew (kurtosis)	5.13 (33.5)	5.9 (48.6)	4.55 (24.7)	4.77 (30.8)	3.23 (15.8)	2.73 (12.5)	1.99 (5.69)	.01 (2.25)
Reported no losses								
*n* (%)	17 (13%)	7 (5%)	12 (16%)	5 (7%)	5 (10%)	2 (4%)	0 (0%)	0 (0%)
PGSI (last year)								
M (SD)	.7 (3.6)	20.1 (4.2)	.5 (2.5)	19.9 (4.5)	1.1 (5.0)	20.3 (4.1)	0 (0)	20.8 (2.9)
NODS (last 30-days)								
M (SD)	–	6.5 (2.2)	–	6.8 (2.4)	–	6.3 (1.8)	–	6.2 (2.1)

NODS, the NORC diagnostic screen for gambling problems; PGSI, Problem Gambling Severity Index; SEK, Swedish Kronor (1 USD = 8.88 SEK); IQR, interquartile range

### Agreement Concerning Money Lost When Gambling

**Fig. 3:**
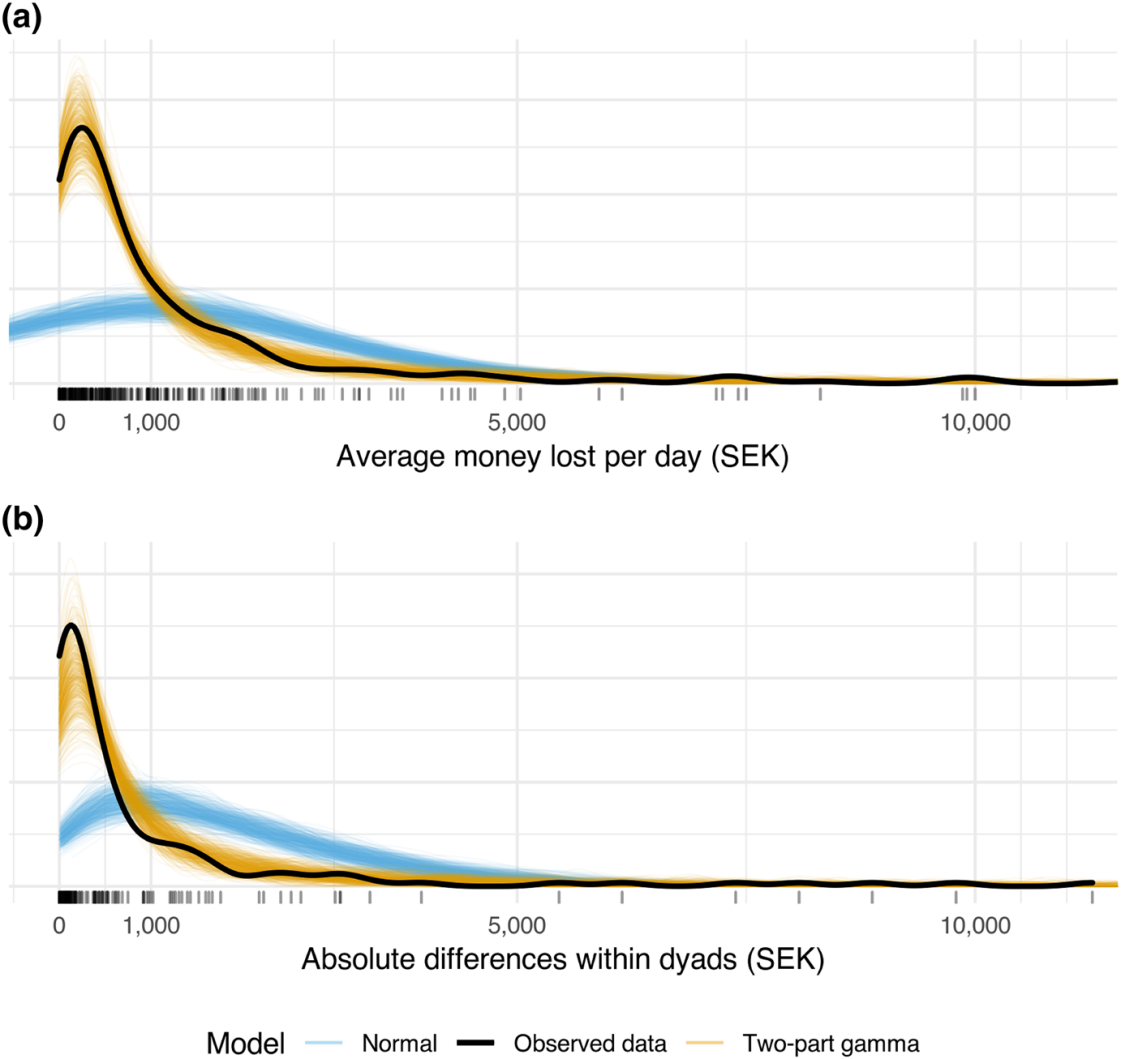
Posterior predictive distribution of the TLFB losses based on either the two-part gamma model or the normal (Gaussian) LMM with the observed data overlaid. The negative predictions from the normal LMM have been truncated. The rug above the *x* axis shows the individual observations. 1 USD = 8.88 SEK (Color figure online)

Both the posterior predictive checks and the 10-fold CV indicate the gamma response distribution provided a better fit than the lognormal distribution, as well as a substantively better fit than the Gaussian distribution, as shown in Table 2. Figure 3 shows the posterior predictive distribution comparing the two-part gamma model to the Gaussian LMM.^4^

**Table 2:** Model comparison using 10-fold cross-validation

	Model comparison	10-fold CV_diff_	SE
ICC overall	Gamma versus Gaussian	− 852.3	78.6
	Gamma versus lognormal	− 33.9	19.9
Relationship	Gamma versus Gaussian	− 1173.6	219.8
	Gamma versus lognormal	− 67.8	34.3

Gamma, two-part gamma GLMM; Lognormal, two-part lognormal GLMM; Gaussian, ordinary LMM; 10-fold CV_diff_, difference between the models’ predictive information criteria from a 10-fold cross validation; SE, standard error of 10-fold CV_diff_

Overall, there was a fair level of agreement^5^ between the CSOs and the gamblers concerning the amount of money lost when gambling, as shown in Table 3. Table 4 breaks down the results per CSO type and illustrates that the reports obtained from CSOs who were partners tended to exhibit better agreement with the gamblers’ reports when compared to the reports obtained from CSOs who were parents and CSOs who were neither parents nor partners. The difference between the partner CSOs and the parent CSOs was most clear for the losses part, with a smaller difference being seen in the level of agreement regarding the overall reports. However, all the models had point estimates that indicated that the partner CSOs exhibited better agreement than the other CSOs, and the findings based on Spearman’s rho in Table 5 show the same result.

**Table 3:** TLFB overall agreement for the different response distributions

Distribution	ICC					
	Overall	95% CI	Losses	95% CI	Zero losses	95% CI
Two-part gamma (Indep)	.53	(.45, .61)	.57	(.48, .64)	.40	(.16, .62)
Two-part gamma (corr)	.57	(.48, .64)	.58	(.50, .65)	.39	(.15, .61)
Two-part lognormal (Indep)	.34	(.23, .43)	.36	(.24, .46)	.40	(.17, .62)
Two-part lognormal (corr)	.36	(.25, .46)	.36	(.25, .47)	.39	(.15, .61)
Gaussian	.76	(.67, .82)	.75	(.66, .83)	–	–

Overall, includes both no losses (zeros) and losses; Losses, includes only losses (TLFB > 0); zero losses, binary part of the model; Indep, independent random effects; Corr, correlated random effects in the two-part GLMM model

**Table 4:** TLFB agreement per CSO relationship type, from the two-part gamma GLMM

CSO type	ICC						
	Model	Overall	95% CI	Losses	95% CI	Zero losses	95% CI
Parent	Indep	.39	(.21, .53)	.42	(.22, .56)	.25	(.00, .67)
	Corr	.45	(.28, .59)	.46	(.28, .59)	.36	(.03, .70)
Partner	Indep	.61	(.51, .69)	.65	(.55, .73)	.52	(.23, .74)
	Corr	.62	(.45, .71)	.65	(.55, .73)	.52	(.22, .75)
Other	Indep	.46	(.05, .73)	.46	(.05, .74)	.18	(.00, .91)
	Corr	.45	(.03, .73)	.46	(.02, .74)	.20	(.00, .90)
Differences							
Partner–parent	Indep	.21	(.04, .42)	.23	(.06, .45)	.25	(− .23, .65)
	Corr	.16	(− .05, .36)	.20	(.03, .39)	.16	(− .30, .57)
Partner–other	Indep	.15	(− .15, .55)	.19	(− .11, .60)	.29	(− .49, .71)
	Corr	.16	(− .16, .58)	.19	(− .10, .63)	.27	(− .51, .71)

Overall, includes both no losses (zeros) and losses; Losses, includes only losses (TLFB > 0); Zero losses, binary part of the model; Indep, independent random effects; Corr, correlated random effects in the two-part GLMM model; Other, CSOs who were neither parent nor partner

**Table 5:** Correlation between gamblers’ and CSOs’ reports

	Spearman’s rho
Overall	.64
Parent	.56
Partner	.66
Other	.13

The two-part gamma GLMM was also used to investigate whether there were any systematic differences in the CSOs’ and the gamblers’ reports. The results presented in Table 6 show that a larger proportion of the CSOs reported no losses when compared to the gamblers (14% vs. 6%).

**Table 6:** The proportion of CSOs and gamblers who reported zero losses

	Proportion reporting zero losses	95% CI
CSOs	.14	(.09, .21)
Gamblers	.06	(.03, .11)
Diff (CSOs–Gamblers)	.08	(.03, .14)

Table 7 shows that the results were inconclusive with regard to the existence of any systematic differences in the average daily losses, with no indication of any large differences being found. Furthermore, within almost half of the dyads, the CSOs reported higher losses than their respective gamblers. Indeed, the proportion of CSOs with higher reports was .43, 95% CI (.34, .52).

**Table 7:** Average differences between CSOs and gamblers in the overall reported daily losses

	Daily losses (marginal mean, SEK)	95% CI
CSOs	1381	(1013, 2006)
Gamblers	1425	(1055, 2044)
Diff (CSO–Gambler)	− 46	(− 370, 283)
Ratio (CSO/Gambler)	.97	(.78, 1.21)

SEK, Swedish kronor (1 USD = 8.88 SEK)

### Gambling Debt and Years Spent Gambling

Overall, there was excellent agreement in terms of the years spent gambling, ICC = .79, 95% CI (.71, .85), with equal point estimates being found for parents and partners, ICC = .76 versus .76, albeit with the 95% CI (− .16, .17) of the difference indicating that important differences could not be ruled out. There was fair agreement overall regarding the amount of gambling debt, ICC = .57, 95% CI (.46, .65). However, the parent CSOs showed higher agreement when compared to the partner CSOs, ICC_*diff*_ = − .39, 95% CI (− .62, − .17), with more inconclusive results being found when comparing to the CSOs who were neither partners nor parents, ICC_*diff*_ = − .30, 95% CI (− .60, .32). The results for both years gambling and gambling debt are shown in Table 8.

**Table 8:** Agreement between the CSOs and gamblers on years spent gambling and gambling debt, both per relationship type and overall

	ICC			
	Years gambling	95% CI	Gambling debt	95% CI
Overall	.79	(.71, .85)	.57	(.46, .65)
Per relation				
Parent	.76	(.62, .87)	.75	(.61, .82)
Partner	.76	(.65, .86)	.36	(.14, .53)
Other	.92	(.78, .99)	.67	(.07, .85)
Differences				
Parent–partner	.001	(− .16, .17)	− .39	(− .62, − .17)
Parent–other	− .15	(− .29, .01)	− .30	(− .60, .32)

ICC, intraclass correlation

### Sensitivity Analyses

Sensitivity analyses were performed to check whether the results were impacted by (1) excluding those dyads with a perfect agreement, (2) including dyads with sign up dates differing by more than 7 days, and (3) including only those dyads with a maximum absolute difference in sign-up dates of 1 day. None of these model decisions had a major impact on the results. The sensitivity analyses are detailed in the online appendix.^6^

### Simulation Results

**Fig. 4:**
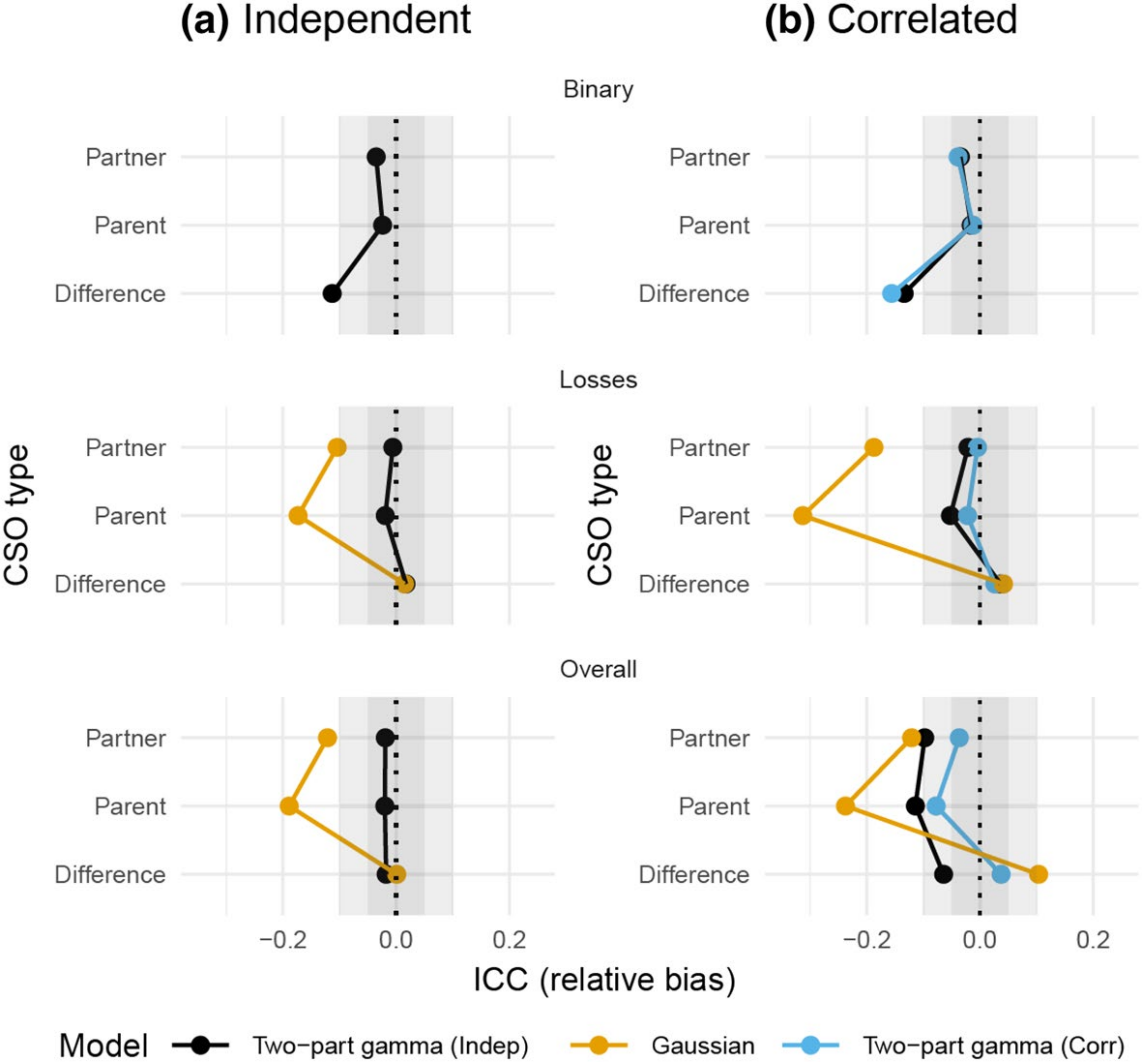
Relative bias of the ICCs obtained from the simulations: **a** results when the true model included no correlation between the random effects, while **b** results when the random effects in the two-part model were correlated (Color figure online)

**Fig. 5:**
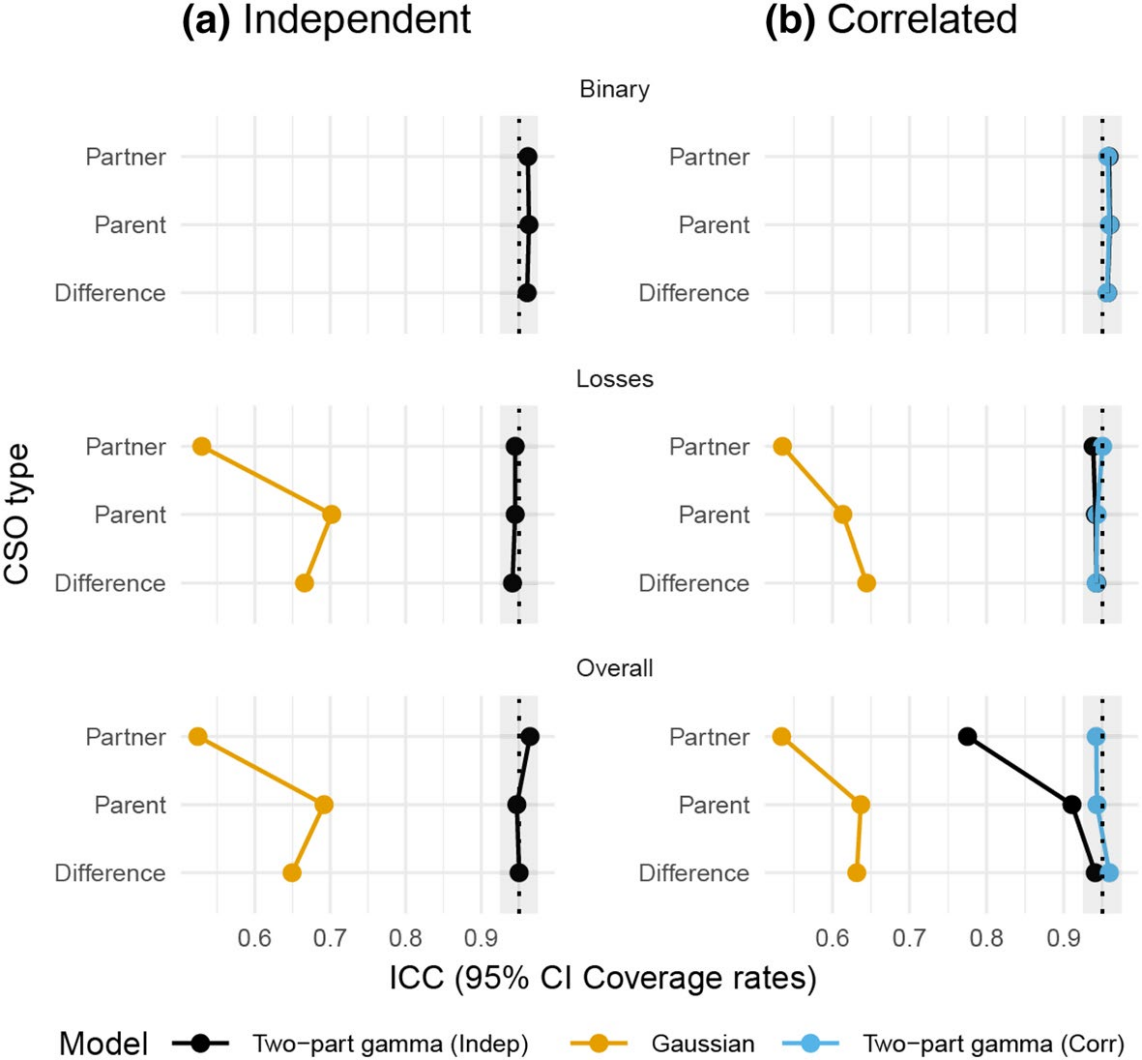
Coverage rates for the 95% CIs from the simulations: **a** results when the true model included no correlation between the random effects, while **b** results when the random effects in the two-part model were correlated (Color figure online)

Figures 4 and 5 show that, although the relative bias for the ICCs obtained from the Gaussian model was seldom more than ± 20%, the estimates exhibited low precision, and the 95% CIs had coverage probabilities much smaller than .95, whereas the two-part gamma GLMM yielded both low relative bias and appropriate coverage probabilities. As anticipated, the binary part of the model showed some bias, although the CIs had good coverage rates. This only had a minor impact on the ICCs for the overall outcomes. Ignoring the true correlation between the random effects in the two-part model led to increased bias and worse coverage rates for the overall ICCs, although it is worth noting that the inferences regarding the ICCs for only the losses part of the two-part model worked well in all the scenarios.

**Fig. 6:**
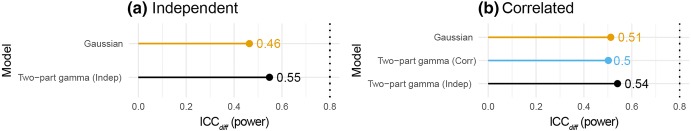
Empirical power to detect a difference between the ICCs obtained from partner CSOs and parent CSOs: **a** results when the true model included no correlation between the random effects, while **b** results when the random effects in the two-part model were correlated (Color figure online)

The power to detect a difference between the ICCs of the partner CSOs and the parent CSOs was slightly higher for the two-part gamma GLMM when compared to the Gaussian LMM, as shown in Fig. 6.

## Discussion

The aim of this study was twofold. First, to investigate the level of agreement between gamblers’ and CSOs’ reports of the amount of money lost when gambling, as well as to determine whether the type of CSO relationship affects the level of agreement. Second, to investigate if there are better alternatives than assuming a normal distribution when modeling the level of agreement.

The results showed that the CSOs’ assessment of the amount of money lost due to gambling exhibited fair agreement with the respective gambler’s report, which is in line with the findings of previous research (Hodgins and Makarchuk 2003). However, given that the present study’s design could potentially lead to circumstances in which the CSOs have more knowledge than usual regarding the gambling problem, it must be recognized that the estimates are still quite far from being concordant. The gamblers and the CSOs signed up for this study together, and they are likely to have at least touched upon the subject before or while signing up. The CSOs could simply have asked about the gamblers’ gambling patterns. Moreover, a large portion of the participating gamblers gambled online, which makes records of their gambling relatively easily accessible, either through the gambling companies’ websites or through bank account details. In studies involving only CSOs of treatment-refusing gamblers (Hodgins et al. 2007; Magnusson et al. 2015) knowledge of the gambling problem is likely to be lower.

The results indicated no large systematic differences between the reports obtained from the CSOs and those obtained from the gamblers, with close to half of the CSOs reporting lower losses than their respective gambler.

The results exhibited a certain degree of conflict regarding any differences between the partner CSOs and the parent CSOs, which was made even more complex by the small proportion of participants who reported zero losses. Although the point estimates in all the models indicated that the partner CSOs generally exhibited a higher level of agreement than the parent CSOs, the CIs were close to zero, and in one case included zero. This meant that even the CIs that indicated a non-zero difference did not exclude a difference small enough to be meaningless. However, there are several reasons why partner CSOs would provide better reports concerning the amount of money lost when gambling. For instance, on average the partner CSOs saw the respective gambler almost every day, as compared to only every second day for the parent CSOs. This is in line with similar research conducted in the field of problem drinking, where the days spent together were related to the accuracy of the CSOs’ reports. For example, Connors and Maisto (2003) found that the correlation between the reports provided by naturally recovered subjects and a spouse or partner was .73, while it was .27 when the collateral was a non-spouse or partner. It is reasonable to assume that many partners have shared finances, which increases their insight into each other’s financial situation (Gunnarsson and Wahlund 1997; Heimdal and Houseknecht 2003). Moreover, the qualitative assessment conducted as part of the pilot testing of the intervention evaluated in the RCT revealed that some gamblers, especially in parent–child dyads, appear to have been pressured into participation by their CSO (Nilsson et al. 2018). This could affect the gambler’s willingness to reveal details about his or her gambling. Finally, the gamblers in the parent–child dyads tended to be younger, often in their late twenties, while the gamblers with partner CSOs were, on average, 10 years older.

In terms of the gambling-related debt, the level of agreement was fair overall; however, the partner CSOs’ reports showed much lower agreement when compared to the parent CSOs’ reports and the reports of the CSOs who were neither parents nor partners, whom all offered reports that exhibited good agreement. The reports concerning the years of problem gambling exhibited good agreement both overall and per relationship type. It is unsurprising that the level of agreement tended to be higher for the years spent gambling since the range of possible outcomes is much narrower when compared to the gambling losses or gambling debt.

It should be noted that the gamblers themselves might have limited insight into the extent and nature of their own gambling. Several prior studies have revealed that there is an overall discrepancy between gamblers’ accounts of their gambling and the overall gambling turnover reported by gambling corporations (Blaszczynski et al. 2008; Williams and Wood 2004). There has also been studies pointing to the possibility of a tendency among problem gamblers to underreport their spending on gambling due to social desirability (Goldstein et al. 2017). This has prompted researchers to assume that gamblers, in general, tend to underestimate their losses. However, studies comparing self-reports with actual individual gambling outcomes on online gambling sites paint a somewhat different picture. For instance, Braverman et al. (2014) found that only 10% of the 2259 surveyed gamblers could accurately recall both their wins and their losses, while 40% had a positive bias of the outcomes. Gambling problems were associated with particularly low accuracy in terms of self-reports, although participants with gambling problems were more likely to estimate their outcomes unfavorably than favorably. A study by Auer and Griffiths (2017) found that the estimated losses and the actual losses were correlated, although the gamblers tended to overestimate their wins and underestimate their losses. The study also showed that gamblers who gambled more intensely were less accurate in terms of their self-reports.

To the best of our knowledge, this is the first study to account for the distributional characteristics of gambling data when modeling the level of agreement. Our results highlight the importance of model assumptions when working with gambling losses, in our study, the ICCs based on the normal distributions were much higher than the more appropriate gamma or lognormal distributions. The two-part GLMM used in this study is much more complicated than the Gaussian LMM. However, both our analyses using real data and our simulation indicate that the assumption of normality is highly unlikely to hold in the case of reports of gambling losses. Further, the study shows that assuming normality will lead to unreliable estimates of the agreement between gamblers and CSOs, with CIs that have low coverage probabilities and estimates that exhibit low precision. In this study, a two-part gamma GLMM with a log-link seemed to work well, and the simulation results showed that the model had equal or better power when compared to the Gaussian model, while simultaneously providing a much richer answer.

## Limitations

Self-report measures of gambling losses will always involve a certain degree of error, and the loss of money might be difficult to recall during particularly intense periods of gambling. Some CSOs reported that they had no information at all regarding past gambling, and thus the information they provided is likely to be inaccurate. Furthermore, it was not record whether the gambler and the CSO cohabitate only how many days per week they spent together. Similarly, it was not investigated how much of the differences in the level of agreement between CSO types could be explained by other variables, such as cohabitating and age. Future studies could investigate if such variables are better markers then the CSO’s relationship to the gambler. Lastly, the performance of the two-part model was only evaluated in a limited set of scenarios. Future research studies should investigate the ICC estimates when the model is misspecified, for example, when the response distribution is incorrect.

## Conclusions

The results of this study show that CSOs tend to exhibit moderately good insight into the amount of money lost due to gambling, a fair degree of knowledge concerning gambling debt, and an excellent insight into the years of problem gambling. Partner CSOs seem to have better knowledge regarding the amount of money lost due to gambling when compared to parent CSOs, while parent CSOs have a more accurate understanding of the level of gambling-related debt than partner CSOs. Furthermore, this study shows that when assessing agreement in terms of gambling losses, it is important to use a model that allows for the distribution of the reports to be skewed, while still including reports that are zero. Using the normal distribution when modeling the level of agreement concerning money lost due to gambling (or similar skewed outcomes) produces results that are likely to be highly unreliable.

## Footnotes

1 The full list of inclusion criteria can be found in the study protocol (Nilsson et al. 2016).

2 Two-part models are also known as hurdle or semicontinuous models.

3 The technical details for this model can be found in Appendix 1 and the code to run the model in Appendix 2 which is available from https://osf.io/ec5b9/.

4 Additional posterior predictive checks can be downloaded from https://osf.io/ec5b9/.

5 The ICCs were interpreting using Cicchetti’s (1994) guidelines: “poor” $\le$ .4, “fair” = .40–.59, “good” = .60–.74, and “excellent” = .75–1.00.

6 The appendix can be downloaded from https://osf.io/ec5b9/.

## References

[CR1] American Psychiatric Association (2013). Diagnostic and statistical manual of mental disorders (DSM-5^®^).

[CR2] Auer M, Griffiths MD (2017). Self-reported losses versus actual losses in online gambling: An empirical study. Journal of Gambling Studies.

[CR3] Blaszczynski A, Ladouceur R, Goulet A, Savard C (2008). Differences in monthly versus daily evaluations of money spent on gambling and calculation strategies. Journal of Gambling Issues.

[CR4] Borsari B, Muellerleile P (2009). Collateral reports in the college setting: A meta-analytic integration. Alcoholism: Clinical and Experimental Research.

[CR5] Braverman J, Tom MA, Shaffer HJ (2014). Accuracy of self-reported versus actual online gambling wins and losses. Psychological Assessment.

[CR6] Bürkner P-C (2017). brms: An R package for Bayesian multilevel models using Stan. Journal of Statistical Software.

[CR7] Carlbring P, Smit F (2008). Randomized trial of internet-delivered self-help with telephone support for pathological gamblers. Journal of Consulting and Clinical Psychology.

[CR8] Carpenter B, Gelman A, Hoffman M, Lee D, Goodrich B, Betancourt M, Guo J, Li P, Riddell A (2017). Stan: A probabilistic programming language. Journal of Statistical Software, Articles.

[CR9] Cicchetti DV (1994). Guidelines, criteria, and rules of thumb for evaluating normed and standardized assessment instruments in psychology. Psychological Assessment.

[CR10] Connors GJ, Maisto SA (2003). Drinking reports from collateral individuals. Addiction.

[CR11] de Villemereuil P, Schielzeth H, Nakagawa S, Morrissey M (2016). General methods for evolutionary quantitative genetic inference from generalized mixed models. Genetics.

[CR12] Dickson-Swift VA, James EL, Kippen S (2005). The experience of living with a problem gambler: Spouses and partners speak out. Journal of Gambling Issues.

[CR13] Diskin KM, Hodgins DC (2009). A randomized controlled trial of a single session motivational intervention for concerned gamblers. Behaviour Research and Therapy.

[CR14] Downs C, Woolrych R (2010). Gambling and debt: The hidden impacts on family and work life. Community, Work and Family.

[CR15] Gelman A, Carlin JB, Stern HS, Dunson DB, Vehtari A, Rubin DB (2014). Bayesian data analysis.

[CR16] Gelman A, Meng X-L, Stern H (1996). Posterior predictive assessment of model fitness via realized discrepancies. Statistica Sinica.

[CR17] Goldstein AL, Vilhena-Churchill N, Munroe M, Stewart SH, Flett GL, Hoaken PNS (2017). Understanding the effects of social desirability on gambling self-reports. International Journal of Mental Health and Addiction.

[CR18] Graham K, Braun K (1999). Concordance of use of alcohol and other substances among older adult couples. Addictive Behaviors.

[CR19] Gunnarsson J, Wahlund R (1997). Household financial strategies in Sweden: An exploratory study. Journal of Economic Psychology.

[CR20] Hagman BT, Cohn AM, Noel NE, Clifford PR (2010). Collateral informant assessment in alcohol use research involving college students. Journal of American College Health.

[CR21] Heimdal KR, Houseknecht SK (2003). Cohabiting and married couples’ income organization: Approaches in Sweden and the United States. Journal of Marriage and Family.

[CR22] Hodgins DC, Currie SR, Currie G, Fick GH (2009). Randomized trial of brief motivational treatments for pathological gamblers: More is not necessarily better. Journal of Consulting and Clinical Psychology.

[CR23] Hodgins DC, Makarchuk K (2003). Trusting problem gamblers: Reliability and validity of self-reported gambling behavior. Psychology of Addictive Behaviors.

[CR24] Hodgins DC, Toneatto T, Makarchuk K, Skinner W, Vincent S (2007). Minimal treatment approaches for concerned significant others of problem gamblers: A randomized controlled trial. Journal of Gambling Studies.

[CR25] Holdsworth L, Nuske E, Tiyce M, Hing N (2013). Impacts of gambling problems on partners: Partners’ interpretations. Asian Journal of Gambling Issues and Public Health.

[CR26] Kalischuk RG, Nowatzki N, Cardwell K, Klein K, Solowoniuk J (2006). Problem gambling and its impact on families: A literature review. International Gambling Studies.

[CR27] Kenny DA, Kashy DA, Cook WL, Simpson J (2006). Dyadic data analysis.

[CR28] Laforge RG, Borsari B, Baer JS (2005). The utility of collateral informant assessment in college alcohol research: Results from a longitudinal prevention trial. Journal of Studies on Alcohol.

[CR29] Liu L, Strawderman RL, Cowen ME, Shih Y-CT (2010). A flexible two-part random effects model for correlated medical costs. Journal of Health Economics.

[CR30] Magnusson K, Nilsson A, Hellner Gumpert C, Andersson G, Carlbring P (2015). Internet-delivered cognitive-behavioural therapy for concerned significant others of people with problem gambling: Study protocol for a randomised wait-list controlled trial. British Medical Journal Open.

[CR31] Maisto SA, Sobell LC, Sobell MB (1979). Comparison of alcoholics’ self-reports of drinking behavior with reports of collateral informants. Journal of Consulting and Clinical Psychology.

[CR32] Makarchuk K, Hodgins DC, Peden N (2002). Development of a brief intervention for concerned significant others of problem gamblers. Addictive Disorders and Their Treatment.

[CR33] McBride J, Derevensky J (2008). Internet gambling behavior in a sample of online gamblers. International Journal of Mental Health and Addiction.

[CR34] Neelon B, O’Malley AJ, Smith VA (2016). Modeling zero-modified count and semicontinuous data in health services research Part 1: Background and overview. Statistics in Medicine.

[CR35] Nilsson A, Magnusson K, Carlbring P, Andersson G, Hellner Gumpert C (2016). Effects of added involvement from concerned significant others in internet-delivered CBT treatments for problem gambling: Study protocol for a randomised controlled trial. British Medical Journal Open.

[CR36] Nilsson A, Magnusson K, Carlbring P, Andersson G, Hellner Gumpert C (2018). The development of an internet-based treatment for problem gamblers and concerned significant others: A pilot randomized controlled trial. Journal of Gambling Studies.

[CR37] Olsen MK, Schafer JL (2001). A two-part random-effects model for semicontinuous longitudinal data. Journal of the American Statistical Association.

[CR38] Patford J (2007). For poorer: How men experience, understand and respond to problematic aspects of a partner’s gambling. Gambling Research: Journal of the National Association for Gambling Studies (Australia).

[CR39] Patford J (2009). For worse, for poorer and in ill health: How women experience, understand and respond to a partner’s gambling problems. International Journal of Mental Health and Addiction.

[CR40] Petry NM, Ammerman Y, Bohl J, Doersch A, Gay H, Kadden R, Molina C, Steinberg K (2006). Cognitive-behavioral therapy for pathological gamblers. Journal of Consulting and Clinical Psychology.

[CR41] Public Health Agency of Sweden. (2016). *Tabellsammanställning för Swelogs prevalensstudie 2015. Med jämförelser mot prevalensstudien 2008/2009. [Compilation of the Swelogs Prevalence Study 2015. With comparisons to the prevalence study 2008/2009]* (No. 16139). Retrieved from https://www.folkhalsomyndigheten.se/globalassets/livsvillkor-levnadsvanor/andts/spel/swelogs/tabellsammanstallning-swelogs-prevalensstudie-2015.pdf. Accessed 20 Sept 2018.

[CR42] R Core Team. (2018). *R: A language and environment for statistical computing*. Vienna: R Foundation for Statistical Computing. Retrieved from https://www.R-project.org/. Accessed 20 Sept 2018.

[CR43] Sobell LC, Agrawal S, Sobell MB (1997). Factors affecting agreement between alcohol abusers’ and their collaterals’ reports. Journal of Studies on Alcohol.

[CR44] Stasiewicz PR, Stalker RG (1999). Subject-collateral reports of drinking in inpatient alcoholics with comorbid cocaine dependence. The American Journal of Drug and Alcohol Abuse.

[CR45] Svensson J, Romild U, Shepherdson E (2013). The concerned significant others of people with gambling problems in a national representative sample in Sweden—A 1 year follow-up study. BMC Public Health.

[CR46] Taber JI, McCormick RA, Russo AM, Adkins BJ, Ramirez LF (1987). Follow-up of pathological gamblers after treatment. The American Journal of Psychiatry.

[CR47] Vehtari A, Gelman A, Gabry J (2017). Practical Bayesian model evaluation using leave-one-out cross-validation and WAIC. Statistics and Computing.

[CR48] Walker M, Toneatto T, Potenza MN, Petry N, Ladouceur R, Hodgins DC, El-Guebaly N, Echeburua E, Blaszczynski A (2006). A framework for reporting outcomes in problem gambling treatment research: The Banff, Alberta Consensus. Addiction.

[CR49] Weinstock J, Whelan JP, Meyers AW (2004). Behavioral assessment of gambling: An application of the timeline followback method. Psychological Assessment.

[CR50] Williams RJ, Wood RT (2004). The demographic sources of Ontario gaming revenue.

